# hsa-miR-548d-3p: a potential microRNA to target nucleocapsid and/or capsid genes in multiple members of the Flaviviridae family 

**DOI:** 10.3389/fbinf.2024.1487292

**Published:** 2025-01-14

**Authors:** H. W. Cayatineto, S. T. Hakim

**Affiliations:** Hakim’s Lab, Department of Biology, School of STEM, Diné College, Tuba City, AZ, United States

**Keywords:** *Flavivirus*, miRNA, BLAST, NCBI, alignments, antiviral

## Abstract

**Introduction:**

Flaviviridae comprise a group of enveloped, positive-stranded RNA viruses that are mainly transmitted through either mosquitoes or tick bites and/or contaminated blood, blood products, or other body secretions. These viruses cause diseases ranging from mild to severe and are considered important human pathogens. MicroRNAs (miRNAs) are non-coding molecules involved in growth, development, cell proliferation, protein synthesis, apoptosis, and pathogenesis. These small molecules are even being used as gene suppressors in antiviral therapeutics, inhibiting viral replication. In the current study, we used bioinformatic tools to predict a possible miRNA sequence that could be complementary to the nucleocapsid (NP) and/or capsid (CP) gene of the Flaviviridae family and provide an inhibitory solution.

**Methods:**

Bioinformatics is a field of science that includes tremendous computational analysis, logarithms, and sequence alignments. To predict the right alignments between miRNA and viral mRNA genomes, we used computational databases such as miRBase, NCBI, and Basic Alignment Search Tool–nucleotides (BLAST-n).

**Results:**

Of the 2,600 mature miRNAs, hsa-miR-548d-3p revealed complementary sequences with the flavivirus capsid gene and bovine viral diarrhea virus (BVDV) capsid gene and was selected as a possible candidate to inhibit flaviviruses.

**Conclusion:**

Although more detailed *in vitro* and *in vivo* studies are required to test the possible inhibitory effects of hsa-miR-548d-3p against flaviviruses, this computational study may be the first step to study further, developing a novel therapeutic for lethal viruses within the Flaviviridae family using suggested candidate miRNAs.

## Introduction

**FIGURE 1:**
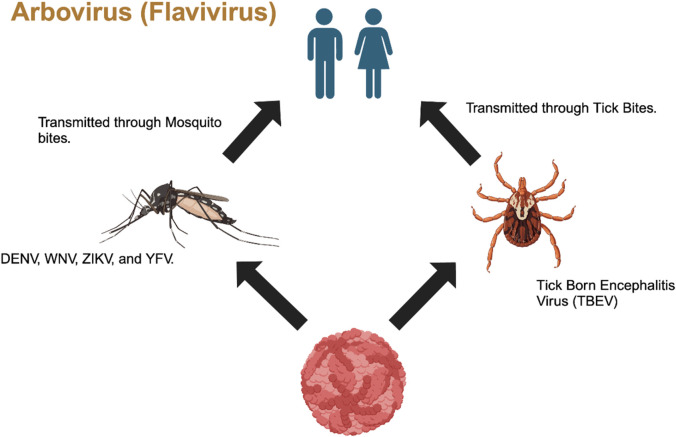
Transmission of Flavivirus from mosquitos and ticks to humans through vector bites (Generated by BioRender).

Arboviruses (arthropod-borne viruses) are a group of viruses that are classified into different taxonomic families such as Flaviviridae, Bunyaviridae, Togaviridae, Rhabdoviridae, Reoviridae, and Asfarviridae, with Flaviviridae, Togaviridae, and Bunyaviridae being the families that cause disease in humans (Giménez-Richarte et al., 2022). In this study, we primarily focused on the Flaviviridae family, which includes four species of pestiviruses, namely, bovine viral diarrhea viruses 1 and 2 (BVDV 1 and BVDV2), classical swine fever virus (CSFV), and border disease virus (BDV) (Mari et al., 2016; Maurer et al., 2004; Warrener and Collett, 1995; Schweize and Peterhans, 2001). The *Flavivirus* genus also includes global human pathogens such as Zika virus (ZIKV), West Nile virus (WNV), Japanese encephalitis virus (JENV), dengue virus (DENV), yellow fever virus (YFV), and tick-borne encephalitis virus (TBEV), which all pose a threat to global public health (Hu et al., 2021; Reed et al., 1998). *Hepacivirus*, another member of the flavivirus family, or hepatitis C virus or simply HCV, is responsible for non-A and non-B hepatitis among humans (Harada et al., 2000; Merwaiss et al., 2019; Suzich et al., 1993). This genus also includes additional 50 arthropod-borne viruses, which are mainly transmitted via mosquito bites and tick bites (Barrows et al., 2018) (Figure 1). Although the primary vectors are mainly mosquitos and ticks, these viruses have also been detected and isolated from bats and rodents (Junglen et al., 2009).

The incubation period of flavivirus infections in humans can range from 3 to 6 days (Conde et al., 2017), with presentation of acute flavivirus diseases ranging from being mild to severe and can be life-threatening (Pierson and Diamond, 2020). Pierson and Diamond mentioned that the symptoms of mild illness are mostly similar to flu-like symptoms, which includes asymptomatic infection and/or self-limiting febrile episodes, while severe illness includes hemorrhagic fever, shock syndrome, encephalitis, paralysis, congenital defects, hepatitis, and hepatic failure (2020; Benzarti et al., 2019). Although there are vaccines currently available for most of these viruses, which have also been successful, however, due to re-establishment of vectors, globalization, and urbanizations, epidemics continue to occur, which restricts effectiveness of these vaccines (Julander et al., 2009; de Oliveira Figueiredo et al., 2020; van Leur et al., 2021). People infected with DENV can develop more severe manifestations like dengue hemorrhagic fever (DHF) and dengue shock syndrome (DSS), which includes vascular leakage or hypovolemic shock and coagulopathy, followed by bleeding, organ impartment, and death (Simmons et al., 2012; Conde et al., 2017).

Of these flaviviruses, WNV and JENV are known as neurotropic viruses and cause acute encephalopathy, causing severe neuroinflammation of the central nervous system (CNS) and the blood–brain barrier (Li et al., 2015). In WNV, symptoms include flaccid paralysis, convulsions, cranial neuropathies, optic neuritis, ataxia, stiffness, rigidity spasms, and tremors that might cause long-term neurological changes (World Health Organization, 2019). JENV shows symptoms similar to that of WNV but is rare and has a much higher fatality rate of 30% (World Health Organization, 2019). TBEV, is also a member of the encephalitis virus family like WNV and JENV, but on the contrary, it is not transmitted by mosquitos like the other members of the arbovirus family, rather it is transmitted by infected tick (*Ixodes ricinus*) bites that can spread from animals to humans (Turtle et al., 2012).

**FIGURE 2:**
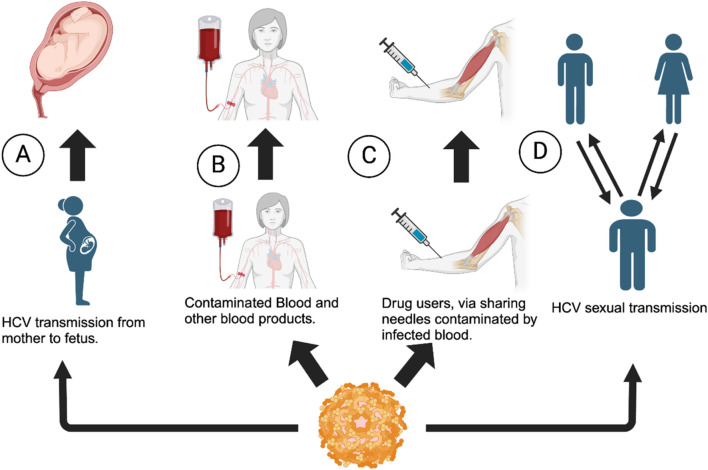
Transmission of HCV virus among individuals. **(A)** From the mother to fetus **(B)** via contaminated blood products, **(C)** via needle sharing among drug abusers and needle injury, and **(D)** via sexual contact: man to man, man to woman, or *vice versa* (Flowchart generated using BioRender).

HCV, another member of flaviviruses, attacks the hepatocytes (liver cells) in humans (Song et al., 2001), causing inflammation of the liver. HCV is a blood-borne virus and is transmitted primarily through infected blood and/or blood products or contaminated body fluids. One example of possible HCV transmission is the sharing of needles among drug abusers who use needle injections. In the mid 2000s, HCV transmission has also occurred among men who have sexual encounters with other men, also known as Men sex with other men (MSM) (Nijmeijer et al., 2019) (Figure 2). Despite treatments currently available, there is no vaccine for HCV (Duncan et al., 2020). When left untreated, HCV can lead to liver cirrhosis and chronic hepatitis C infection, leading to liver carcinoma (Isken et al., 2007). Progression of HCV is rather slow and can remain unnoticed (asymptomatic) for decades until the patient develops liver disease, which results in delay in diagnosis and treatment (Babiker et al., 2017).

YFV infections occur in 12% individuals with a 95% confidence interval and in 5 to 26% individuals with manifestations of jaundice, hemorrhage, and organ failure (Waggoneret al., 2018). Mosquitoes are primary carriers in areas of endemicity and are mainly recorded in Africa and South America, and despite successful vaccinations, outbreaks continue and lead to significant high morbidity and mortality rates (Julander et al., 2009). Julander et al. stated that despite vaccinations, there is a great need for more therapies as there is no antiviral agent available for YFV (2009).

Similar to all flaviviruses, ZIKV is another member, which is transmitted to humans by *Aedes (Stegomyia* subgenus*)* mosquitoes (Hills et al., 2017), and the disease caused by ZIKV can range from mild to severe, with a 3–12-day incubation period (Basarab et al., 2016; Musso and Gubler, 2016). Basarab et al. stated that ZIKV symptoms can include fever, conjunctivitis, arthralgia, myalgia, and itchy rashes (Musso and Gubler, 2016; Musso and Nhan, 2015; Hamel et al., 2015). However, Basarab et al. claimed that symptoms also include headache, retro-orbital pain, peripheral edema, joint pain, and even gastrointestinal disturbances (2016).

**FIGURE 3:**
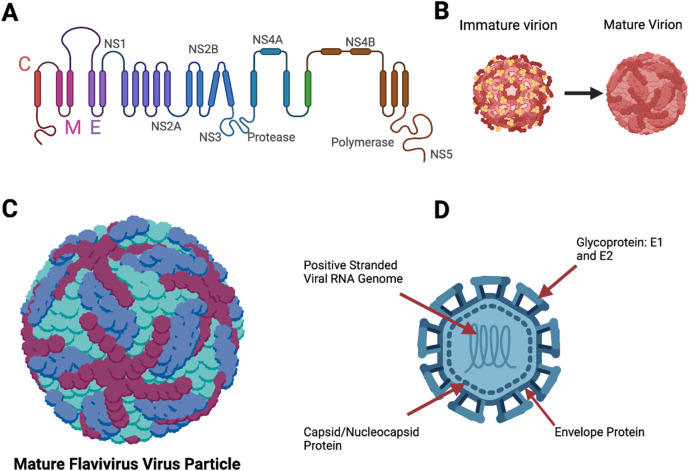
Structures of a flavivirus. **(A)** Polyprotein that makes up most of the viral structure. **(B)** Immature flavivirus particle and a mature flavivirus particle during the replication cycle. **(C)** Mature flavivirus particle with the surface proteins E1 and E2, and envelope structures. E1 is represented in maroon and dark blue colors, and the envelope protein is represented in turquoise color. **(D)** Complete labeled structure of flavivirus, (our target in this study) with the nucleocapsid/capsid protein (Figure 3 was designed using BioRender).

Overall, flaviviruses are small positive-sense, single-stranded RNA viruses that harbor structural proteins such as the capsid (C), which is responsible for protecting the viral genome; the pre-membrane protein (prM); the envelope protein (E); and non-structural (NS) proteins that are categorized as NS2A, NS2B, NS3, NS4A, 2K, NA4B, and NS5 (Mutebi et al., 2004) with a genome of approximately 11 kb (Laureti et al., 2018). During viral entry, replication occurs in the endoplasmic reticulum, where ribosomes are present (Figure 3). Because the genome of these viruses can act as a messenger RNA (mRNA), the genome is readily translated into proteins, making more virus particles (van den Elsen et al., 2021). Viral attachment is accomplished by the E protein attachment to the cognate receptors (Laureti et al., 2018). Laureti et al. conferred that the E protein binds to receptors such as glycosaminoglycans that increase the viral density on the host cell surface, allowing for more effective receptor binding (2018; Perera-Lecoin et al., 2013). On the surface of the E protein, the ectodomain harbors three domains, namely, E-D1, E-2, and E-D3, where E-D3 interacts with attachment factors and receptors and is mainly the target of neutralizing antibodies (Laureti et al., 2018; Pierson Kielian, 2013).

BVDV is a causative agent of bovine diarrhea and mucosal disease and hemorrhagic syndrome with high mortality among cattle (Jackova et al., 2008). The virion size ranges between 40 and 60 nm (Li et al., 2013), and the genome is approximately 13.3 kb in size (Murrayet al., 2008). The viral proteins of BVDV are organized in the following order: NH2-Npro-C-Erns-E1-E2-p7-NS2- NS3-NS4A-NS4B-NS5A-NS5B-COOH (Tellinghuisen et al., 2006; Neill, 2013; Becheret al., 1998; Chi et al., 2022), which is very similar to that of flavivirus polyprotein.

**FIGURE 4:**
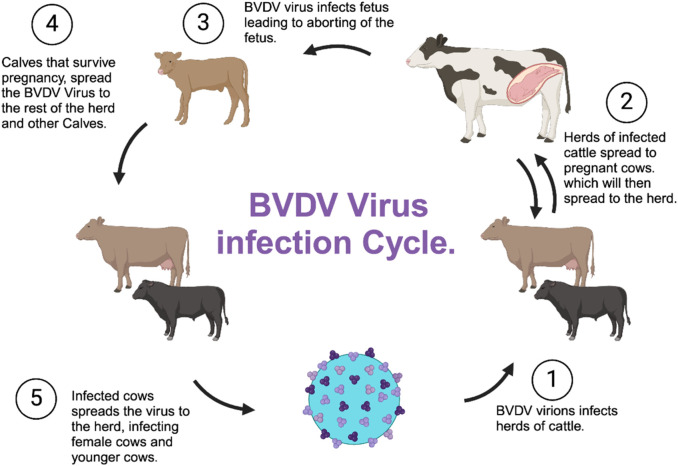
Transmission of the BVDV among a herd of cattle (Generated using BioRender).

Transmission of BVDV among cattle includes fomites, such as contaminated feed, water, and equipment, and among other surfaces such as the nose; tongue; milk bottle nipples; needles; palpitations; secretions; and excretion of urine feces, mucus, milk, and other contaminated materials (Niskanen et al., 2000) (Figure 4). When cattle are exposed, they usually recover over time and shed the virus temporarily; however, pregnant cattle are more susceptible, and the outcome depends on the gestational stage of the fetus (Fulton et al. (2000). Although cows are the main host, BVDV infects various cattle, including bisons, and can cause immune dysfunction and result in asymptomatic infections and seroconversion, including fatal mucosal disease (Hause et al., 2021).

Diseases associated with BVDV can range from clinically inappropriate to severe, even with the availability of vaccines (Xue et al., 2009). Acute and persistent BVDV infections among pregnant cows are often accompanied by transmission into the fetuses, resulting in abortions, teratogenic changes, or delivery of persistently infected, immunotolerant calves, depending on the gestation period (Kosinova et al., 2007). In the transmission process, if a cow is pregnant and is infected with the virus, the virus is transmitted to the fetus (Khodekaram-Tafti and Farjanikish, 2017). The virus has the ability to cause transplacental infection, resulting in different outcomes depending on the stage, which includes fetal death, malformation, acute syndromes of the neonate, immune tolerance, and lifelong viral persistence (Peterhans et al., 2003).

In the 90s, two small RNAs were discovered in *Caenorhabditis elegans* (*C. elegans*); it was later identified that the longer RNA, about 70 nucleotides, was the precursor of shorter RNAs that were about 22 nucleotides, which were classified as microRNAs (miRNAs) due to their short length (Ardekani and Naeini, 2010). miRNAs are small non-coding segments of RNAs that, unlike mRNAs, which encodes proteins, control various levels of important roles such as animal and human growth regulation, development, gene expression, cell proliferation, apoptosis, and even serves as an initiator for protein synthesis (Ardekani and Naeini, 2010; Ranganathan and Sivasankar, 2014; Finnegan and Pasquinelli, 2013; Fu et al., 2013). Most miRNAs are transcribed from DNA sequences into primary miRNAs (pri-miRNAs), then processed into precursor miRNAs (pre-miRNAs), and then into mature miRNAs (Ha and Kim, 2014; O'Brien et al., 2018).

There are three distinct types of miRNAs: small interference RNA (siRNA), RNA interferences (RNAi), and miRNAs (Qian et al., 2022). These molecules not only regulate gene expression or growth and development but can also suppress viral replication by targeting specific genes, resulting in inhibiting viral growth in its host. In the process of suppressing viral replication, mature miRNAs bind to complementary sequences on the 3′ end of the target mRNAs (Skalsky ans Cullen, 2010). Perfect complementarity miRNAs generally lead to potential cleavage of the mRNA genome, while imperfect complementarity results in repression and destabilization or degradation (Skalsky and Cullen, 2010; Baek et al., 2008; Selbach et al., 2008). In this study, we utilize advance bioinformatics tools to identify a possible complementary miRNA sequence to the nucleocapsid (NP) and/or capsid (CP) gene sequences of the flavivirus family.

## Methods

### Collection of *Flavivirus* genome sequences from NCBI

**FIGURE 5:**
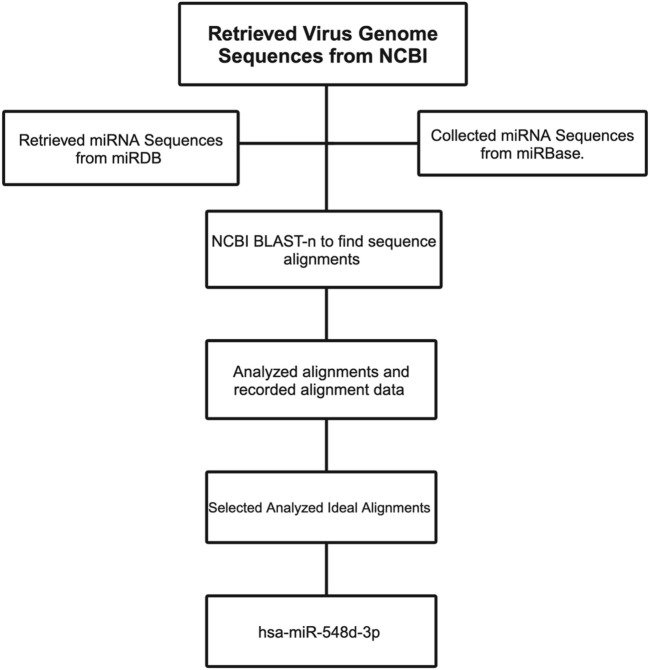
Flowchart demonstrating the workflow to select the right miRNA candidate against the flavivirus genome.

Complementary alignments were carried out using viral genome sequences that are responsible for the nucleocapsid and capsid protein synthesis of BVDV and all flaviviruses and were obtained from the National Center for Biotechnology Information (NCBI) database (Table 1). Figure 5 shows the flowchart of the computational analysis and multiple sequence alignments using miRBase, NCBI, and Basic Local Alignment Search Tool–nucleotides (BLAST-n).

**TABLE 1:** Name of the virus genome, with the accession number, name of genome, and the sequence that were retrieved from the NCBI database.

Virus name	Accession number	Gene name	Sequence
BVDV	AJ715397.1	NP	TCCGACACAAAAGATAAAGGGGCGGTGAGAAAGGAGCAGCAAAAGCCAGATAGGTTGGAAAAGGGGAGAATGAAGATAACACCTAAAGAGTCAGAGAAAGACAGTAAGACCAGGCCACCAGATGCCACAATAGTGGTCGATGGAGTCAAATATCAGGTAAAGAAAAAAGGGAAAGTCAAGAGCAAGAACACCCAGGACGGCTTGTACCACAACAAAAATAAACCTCAAGAGTCTCGCAAGAAACTAGAGAAAGCCCTACTGGCCTGGGCAGTAATAGCCCTGGTTTTGTTTCAAGTC
HCV 1a Strain THCM-NR1/03 capsid protein gene	GQ913857.1	Capsid/core protein	ATGAGCACGAATCCTAAACCTCAAAGAAAAACCAAACGTAACACCAACCGTCGCCCACAGGACGTTAAGTTCCCGGGTGGCGGTCAGATCGTTGGTGGAGTTTACTTGTTGCCGCGCAGGGGCCCTAGATTGGGTGTGCGCGCGACGAGGAAGACTTCCGAGCGGTCGCAACCTCGAGGTAGACGTCAGCCTATCCCCAAGGCGCGTCGGCCCGAGGGCAGGACCTGGGCTCAGCCCGGGTACCCTTGGCCCCTCTATGGCAATGAGGGCTGCGGGTGGGCGGGATGGCTCCTGTCCCCCCGTGGCTCTCGGCCTAGCTGGGGCCCCACAGACCCCCGGCGTAGGTCGCGCAATTTGGGTAAGGTCATCGATACCCTCACGTGCGGCTTCGCCGACCTCATGGGGTACATCCCGCTCGTCGGCGCCCCTCTTGGAGGCGCCGCCAGGGCCCTGGCGCATGGCGTCCGGGTTCTGGAAGACGGCGTGAACTATGCAACAGGGAACCTTCCTGGTTGCTCTTTTTCTATCTTCCTTCTAGCCCTGCTCTCTTGCCTGACTGTGCCCGCGTCAGCC
HCV Genotype 2 isolate MOR34	JN055424.1	Capsid/core protein	ATGAGCACGAATCCTAAACCTCAAAGACAAACCAAAAGAAACACCAACCGCCGCCCAAAGGACGTTAAGTTCCCGGGCGGTGGTCAGATCGTTGGCGGGGTGTACTTGTTGCCGCGCAGGGGCCCTAGATTGGGTGTGCGCGCGACGAGGAAGACCTCGGAGCGATCCCAGCCGCGTGAAAGGCGCCAACCCATCCCCAGGGCTGGGCGCACCACCGGCAGGTCCTGGCAGCAGCCGGGATATCCTTGGCCCCTTTATGGGAACGA
HCV subtype 3a isolate THCM-L3/03	HM042020.1	Capsid/core protein	ATGAGCACACTTCCTAAACCTCAAAGAAAAACCAAAAGAAACACCATCCGTCGCCCACAGGACGTCAAGTTCCCGGGTGGCGGACAGATCGTTGGTGGAGTATACGTGTTGCCGCGCAGGGGCCCACGATTGGGTGTGCGCGCGACGCGTAAAACTTCTGAACGGTCACAGCCTCGCGGACGACGACAGCCTATCCCCAAGGCACGTCGGAGCGAAGGCCGGTCCTGGGCTCAGCCTGGGTACCCTTGGCCCCTCTATGGTAACGAGGGCTGCGGGTGGGCAGGGTGGCTCCTGTCCCCACGCGGCTCCCGTCCATCTTGGGGCCCAAACGACCCCCGGCGACGGTCCCGCAATTTGGGTAAAGTCATCGATACCCTCACATGCGGATTCGCCGACCTCATGGGGTACATCCCGCTCGTCGGCGCTCCCGTAGGGGGCGTCGCAAGGGCCCTCGCACATGGCGTGAGGGCCCTTGAAGACGGGATAAATTTCGCAACAGGGAACTTGCCCGGTTGCTCCTTTTCTATCTTTCTTCTTGCTCTACTCTCTTGCTTAATCCATCCAGCAGCTAGC
HCV type 4 isolate QC27	U33436.1	Capsid/core protein	ATGAGCACGAATCCTAAACCTCAAAGAAAAACCAAACATAACACCAACCGCCGCCCCATGGACGTCAAGTTCCCGGGTGGTGGTCAGATCGTTGGCGGAGTTTACTTGTTGCCGCGCAGGGGCCCTCGTTTGGGTGTGCGCGCGACTCGGAAGACTTCGGAGCGGTCGCAACCTCGTGGGAGACGCCAGCCTATCCCCAAGGCGCGTCGATCCGAGGGAAGGTCCTGGGCACAGCCAGGATACCCATGGCCTCTTTACGGTAATGAGGGTTGCGGGTGGGCAGGATGGCTCCTGTCCCCCCGTGGTTCTCGACCGTCTTGGGGTCCAAATGATCCCCGGCGGAGGTCCCGCAACTTGGGTAAGGTCATCGATACCCTAACCTGCGGCTTCGCCGACCTCATGGGATACATCCCGCTCGTGGGCGCCCCCGTTGGTGGCGTCGCCAGGGCCCTGGCACATGGTGTCAGGGCCGTGGAGGACGGGATTAATTACGCAACAGGGAACCTTCCCGGTTGCTCCTTTTCTATCTTCCTCCTAGCACTCTTTTCGTGCCTGACTGTCCCCGCTTCGGCC
HCV type 5 isolates QC21	U33434.1	Capsid/core protein	ATGAGCACGAATCCTAAACCTCAAAGAAAAACCAAAAGAAACACCAACCGCCGCCCACAGGACGTCAAGTTCCCGGGCGGTGGTCAGATCGTTGGTGGAGTTTACTTGTTGCCGCGCAGGGGCCCTAGGTTGGGTGTGCGCGCGACTCGGAAGACTTCAGAACGGTCGCAACCCCGCGGACGGCGTCAGCCTATTCCCAAGGCGCGCCAATCCGCGGGCCGGTCCTGGGGTCAACCCGGGTACCCTTGGCCCCTTTATGGCAATGAGGGCCTCGGATGGGCAGGGTGGTTGCTCTCCCCCCGGGGTTCTCGGCCTAGTTGGGGCCCCAATGACCCCCGGCGAAAGTCACGTAATTTGGGTAAGGTCATCGATACCCTAACGTGCGGATTCGCCGACCTCATGGGGTATATCCCGCTCGTAGGCGGCCCCGTAGGGGGCGTCGCAAGGGCTCTCGCGCATGGTGTGAGGGTTCTTGAGGACGGGGTAAACTATGCGACAGGGAATTTGCCCGGTTGCTCTTTCTCTATCTTCCTCCTTGCACTTCTCTCGTGCTTGACTGTCCCGGCCTCTGCA
HCV type 6 isolate QC26	U33435.1	Capsid/core protein	ATGAGCACACTTCCAAAACCCCAAAGAAAAACCAAAAGAAACACCAACCGTCGCCCAATGGACGTCAAGTTCCCGGGTGGCGGTCAGATCGTTGGCGGAGTTTACTTGTTGCCGCGCAGGGGCCCACGGTTGGGTGTGCGCGCGACGAGAAAGACTTCCGAGCGATCCCAGCCCAGAGGTAGGCGTCAACCTATACCAAAAGCACGCCAGCCTCAGGGCAGGCACTGGGCTCAGCCCGGATACCCTTGGCCTCTTTATGGAAACGAGGGCTGCGGGTGGGCGGGATGGCTCTTGTCCCCCCGCGGTTCCCGGCCACATTGGGGCCCCAATGACCCCCGGCGTCGATCCCGCAATTTGGGTAAGGTCATCGATACCCTAACGTGTGGATTCGCCGATCTCATGGGGTACATTCCCGTCGTGGGCGCGCCTCTAGGCGGCGTCGCGGCTGCGCTCGCACATGGTGTGAGGGCAATCGAGGACGGGATCAATTATGCAACAGGGAATCTTCCCGGTTGCTCTTTCTCTATCTTCCTTTTGGCACTATTCTCGTGCCTCACGACGCCAGCCTCGGCC
DENV	KM519590.1	Capsid protein	TTCTCAACCGGACTTTTTTCTGGGAAAGGACCCTTACGGATGGTGCTAGCATTCATCACGTTTTTGCGAGTCCTTTCCATCCCACCAACAGCAGGGATTCTGAAAAGATGGGGACAGTTGAAGAAAAATAAGGCCATCAGGATACTGATTGGATTCAGGAAGGAGATAGGCCGCATGCTGAACATCTTGAACGGGAGAAAAAGGTCAACGATAACATTGCTGTGCTTGATTCCCACCGTAATGGCGTTTCACTT
DENV 1	KY346993.1	Capsid protein	ATGAACAACCAACGGAAAAAGACGGGTCGACCGTCTTTCAATATGCTGAAACGCGCGAGAAACCGCGTGTCAACTGGTTCACAGTTGGCGAAGAGATTCTCAAAAGGATTGCTTTCAGGCCAAGGACCCATGAAATTGGTGATGGCTTTCATAGCATTTCTAAGATTTCTAGCCATACCCCCAACAGCAGGAATTTTGGCTAGATGGAGCTCATTCAAGAAGAATGGAGCGATCAAAGTGTTACGGGGTTTCAAAAAAGAGATCTCAAGCATGTTGAACATAATGGATAGAAGGAAAAGA
DENV 2	JQ846016.1	Capsid protein	ATGAATAACCAACGGAAAAAGGCGAAAAACACGCCTTTCAATATGCTGAAACGCGAGAGAAACCGCGTGTCGACTGTGCAACAGCTGACAAAGAGATTCTCACTTGGAATGCTGCAGGGACGAGGACCATTAAAACTGTTCATGGCCCTGGTGGCGTTCCTTCGTTTCCTAACAATCCCACCAACAGCAGGGATATTGAAGAGATGGGGAACAATTAAAAAATCAAAAGCTATTAATGTTTTGAGAGGGTTCAGGAAAGAGATTGGAAGGATGCTGAACATCTTGAATAGGAGACGCAGATCTGCAGGCATGATCATTATGCTGATTCCAACAGTGATGGCG
DENV 3	HQ223036.1	Capsid protein	ATGAACAACCAACGAAAAAAGACGGGAAAACCGTATATCAATATGCTGAAACGCGTGAGAAACCCTGTGTCAACTGGATCACAGTTGGCGAAGAGATTCTCAAGAGGATTGCTCAACGGCCAAGGACCAATGAAATTGGTTATGGCGTTCATAGCTTTCAGATTTCTAGCCATTCCACCAACAGCAGGAGTCTTGGCTAGATGGGGAACCTTCAAGAAGTCAGGGGCTATTAAGGTCCTAAAAGGCTTCAAGAAGGAGATTTCAAACATGCTGAGTATTATCAACAAAAGGAAAAAGACATCGCTCTGTCTCATGATGATGTTACCAGCAACACTTGCT
DENV 4	GQ890685.1	Capsid protein	TTGGTGAAGAGATTCTCAACCGGACTTTTCTCTGGGAAAGGAACCTTACGGATGGTGCTAGCATTCATCACGTTTTTGCGAGTCCTTTCCATCCCGCCAACAGCAGGGATTTTGAAAAGATGGGGACAGTTGAAAAAGAATAAGGCCATCAAGATACTGATTGGATTCAGGAAGGAGATAGGTCGCATGTTAAACATCTTAAATAGGAGAAGAAGGTCAACAATGACATTGCTGTGTTTGATTCCCACCGTAATGGCATTTCACCTGTCAACAAGAGACGGCGAACCCCTCATGATAGTGGCAAAACACGAAAGGGGGAGACCTCTCTTGTTTAAGACAACAGAAGA
JENV	KJ420596.1	Capsid protein	ATCAATATGCTGAAACGCGGCATACCCCGCGTATCCCCACTTGTGGGGGTGAAGAGGGTAATTATGAACTTGCTCGACGGCAGAGGGCCAATACGATTCGTTTTGGCTCTCTTGGCGTTTTTCAAGTTCACAGCACTAGCCCCGACCAAGGCACTCGTTAGCCGATGGAAGGCAGTAGAGAAGAGCGTTGCAATGAAACATCTCACCAGTTTCAAACGAGAACTTGG
YFV	L06480.1	Capsid protein	TCTGGTCGTAAAGCTCAGGGAAAAACCCTGGGCGTCAATATGGTACGACGAGGAGTTCGCTCCTTGTCAAACAAAATAAAACAAAAAACAAAACAAATTGGAAACAGACCTGGACCTTCAAGAGGTGTTCAAGGATTTATCTTTTTCTTTTTGTTCAACATTTTGACTGGAAAAAAGATCACAGCCCACCTAAAGAGGTTGTGGAAAATGCTGGACCCAAGACAAGGCTTGGCTGTTCTAAGGAAAGTTAAGAGAGTGGTGGCCAGTTTAATGAGAGGATTGTCCTCAAGGAAACGCCGTTCCCATGATGTTCTGACTGTGCAATTCCTAATTTTGGGAATGCTGTTGATGACGGGTGGA
WENV	FJ425728.1	Capsid protein	TAACAACAATTAACACAGTGCGAGCTGTTTCTTAGCACGAAGATCTCGATGTCTAAGAAACCAGGAGGGCCCGGTAAAAACCGGGCTGTCAATATGCTAAAACGCGGTATGCCCCGCGGATTGTCCTTGATAGGACTAAAGAGGGCTATGCTGAGTCTGATTGACGGGAAGGGCCCAATACGTTTCGTGTTGGCTCTTTTGGCGTTT
ZIKV	KX443145.1	Capsid protein	TGACAGTTCGAGTTTGAAGCGAAAGCTAGCAACAGTATCAACAGGTTTTATTTTGGATTTGGAAACGAGAGTTTCTGGTCATGAAAAACCCAAAAAAGAAATCCGGAGGATTCCGGATTGTCAATATGCTAAAACGCGGAGTAGCCCGTGTGAGCCCCTTTGGGGGCTTGAAGAGGCTGCCAGCCGGACTTCTGCTGGGTCATGGGCCCATCAGGATGGTCTTGGCGATTCTAGCCTTTTTGAGATTCACGGCAATCAAGCCATCACTGGGTCTCATCAATAGATGGGGTTCAGTGGGGAAAAAAGAGGCTATGGAAATAATAAAGAAGTTCAAGAAAGATCTGGCTGCCATACTGAGAATAATCAATGCTAGGAAGGAGAAGAAGAGACGAGGCGCAGATACTAGTGTCGGAATTGTTGGCCTCCTGCTGACCACAGCTATGGCAGCGGAGGTCACTAGACGTGGGAGTGCATACTATATGTACTTGGACAGAAACGATGCTGGGGAGGCCATATCTTTTCCAACCACATTGGGGATGAATAAGTGTTATATA
TBEV	EU715176.1	Capsid protein	AAATTTTATTACACGCCCAGGGGTTTGCTTCAGACACCAACAGGAGGGCCAGGTTCGGAAGAAACAATCTTTGGTTACTACTAGTCGTGAACGTGTTGAGAAAAAGACAGCTTAGGAGAACAAGAGCTGGGGATGGCCAGGAAGGCCATTCTGAAAGGAAAGGGGGGGGGTCCCCCTCGACAGGTGTCGAAAGGACCCAAAAAGC

### Collection of miRNAs from miRBase and sequence alignments

**FIGURE 6:**
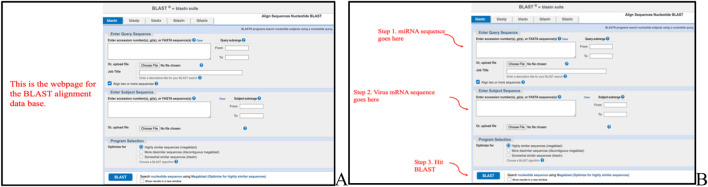
**(A)** Webpage of NCBI BLASTn when finding miRNA–mRNA sequence alignments. **(B)** Method of using BLASTn. The figure demonstrates that the miRNA sequences were entered into the query box and the viral mRNA genome was entered into the subject box.

Figures 8, 6 show the method of predicting the right miRNA sequence using miRBase (https://mirbase.org/) and NCBI BLAST–n) (https://blast.ncbi.nlm.nih.gov/Blast.cgi) (https://blast.ncbi.nlm.nih.gov/Blast.cgi?PROGRAM=blastn&PAGE_TYPE=BlastSearch&LINK_LOC=blasthome). For launching alignments, the miRNA sequences were entered in the Query section of BLAST and the viral mRNA sequence was entered in the Subject section.

## Results

**FIGURE 7:**
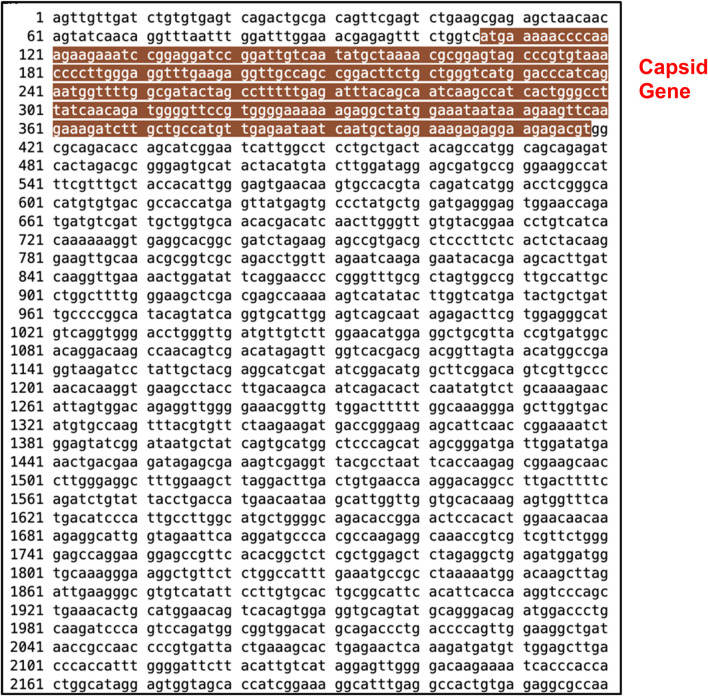
Complete genome sequence of Zika virus retrieved from NCBI GenBank with accession # KX443145.1 with nucleotides 1 through 10,741. The highlighted area indicates the capsid gene at locations 107–418 nucleotides.

**FIGURE 8:**
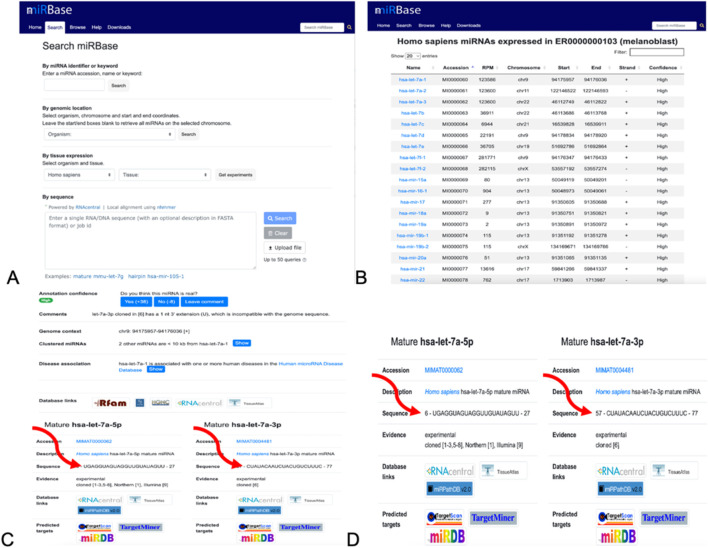
Method of collecting the miRNA sequences from miRBase. **(A)** Homepage. The organism was switched to *Homo sapiens*, exploring the tissues and studying the experiment. **(B)** List of miRNA sequences after performing experiments after selecting the organisms and tissues. **(C)** Result page after performing experiments, where the sequences of the miRNA are indicated by the arrows. **(D)** Information about the mature miRNAs and its sequences. It is these sequences that used using BLASTn.

**FIGURE 9:**
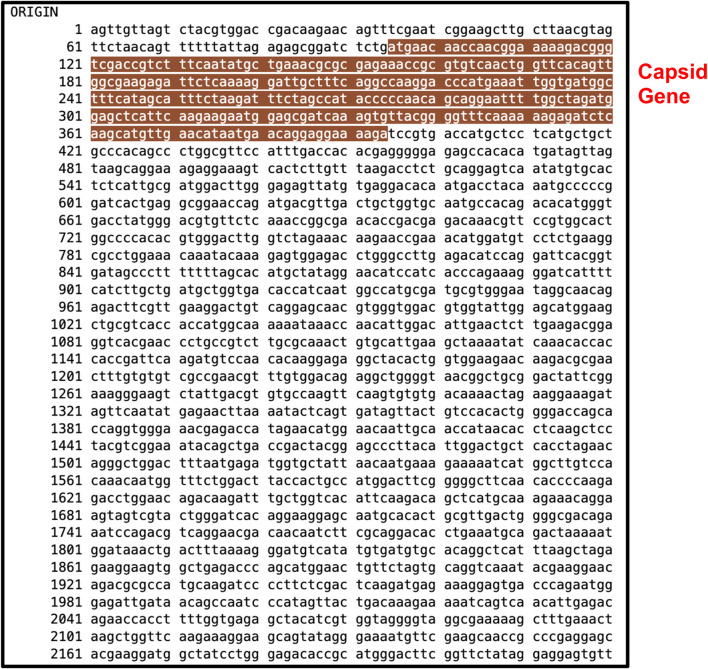
Complete genome sequence of dengue virus retrieved from NCBI GenBank with accession KY346993.1 with nucleotides 1 through 10,681. The highlighted area indicates the capsid gene at locations 95–384 nucleotides.

**FIGURE 10:**
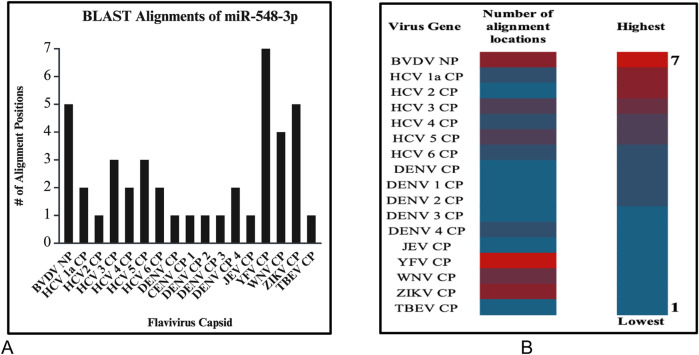
**(A)** Graphs demonstrate the number of perfect alignment position of hsa-miR-548d-3p on the flavivirus capsid genome. The *y*-axis is the number of alignment positions, and the *x*-axis represents the group of viruses in the flavivirus group. **(B)** Heatmap that shows the highest number of predicted miRNA alignment locations (alignment hits) ranging from one to seven locations along the capsid genome of all members of the flaviviruses.

**FIGURE 11:**
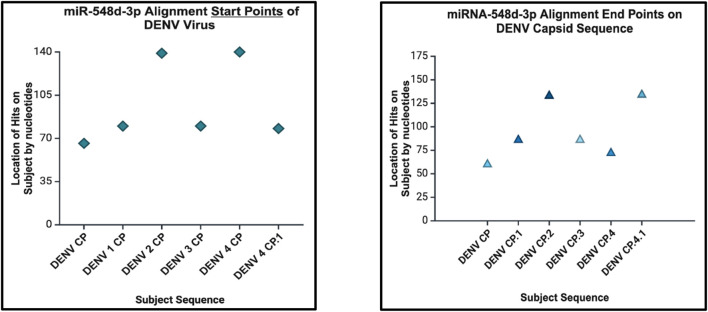
Dot plot that demonstrates the alignment start point (left) and end points (right) of miR-548d-3p on the DENV genome responsible for the capsid protein.

**FIGURE 12:**
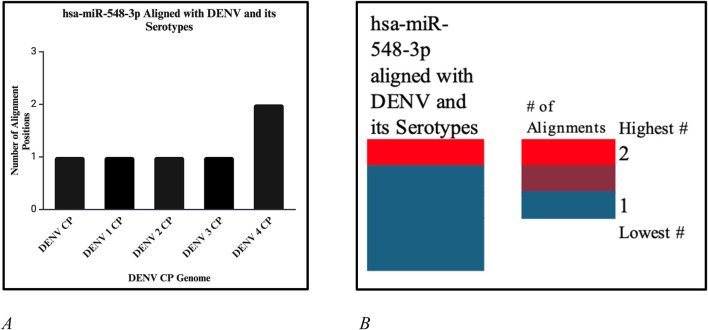
**(A)** Number of alignment positions that hsa-miR-548d-3p reveals on the capsid genome of DENV and its serotypes. On DENV and DENV 1–3, hsa-miR-548d-3p has only 1 alignment position, as compared to DENV 4, where hsa-miR-548d-3p has two alignment locations on the capsid genome. **(B)** Heatmap that shows the highest number of alignment positions between hsa-miR-548d-3p and the capsid genome sequence of the DENV virus and serotypes.

**FIGURE 13:**
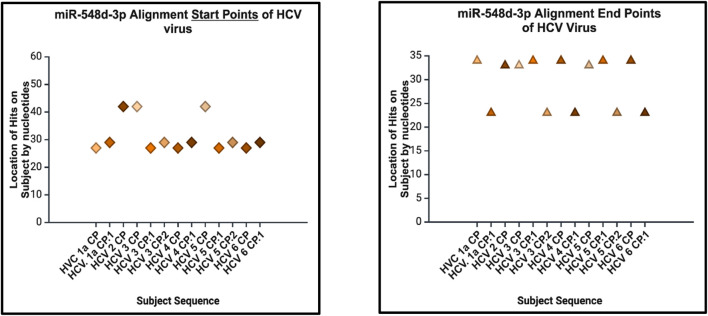
Sequence alignment start points (left) and end points (right) of hsa-miR-548d-3p on the capsid genome of HCV and its genotypes. The location of alignments is indicated on the y-axis, and the viral HCV genome sequences for the capsid protein are indicated on the x-axis. Figure 13 shows that miR-548d-3p has identical matches on identical locations on the genome sequences responsible for the capsid gene on all genotypes of HCV.

**FIGURE 14:**
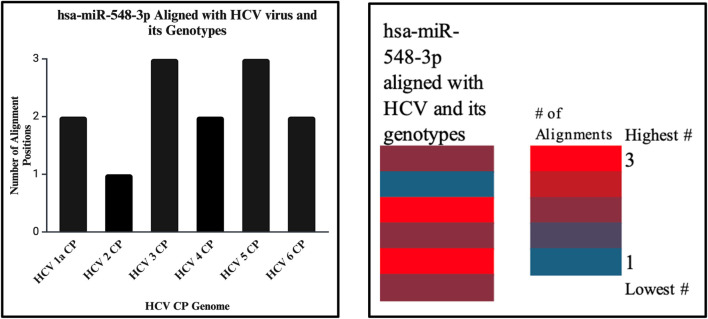
The bar graph (right) shows the highest and lowest number of alignments demonstrated between our miRNA and the genome of the HCV virus. The heatmap (left) shows that the highest number is color coded in red, while the lowest is encoded in blue. In this analysis, both show that the highest number of alignment locations on the HCV virus capsid sequence is 3.

**FIGURE 15:**
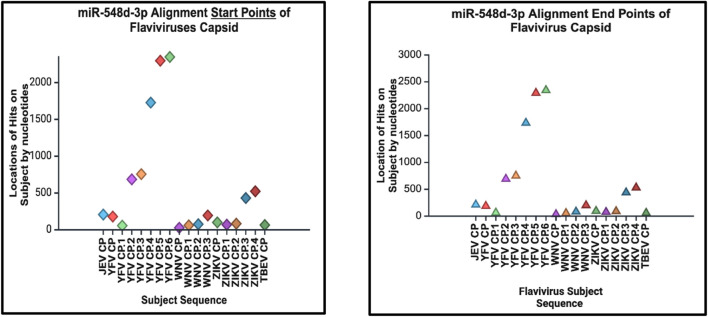
Alignment start points (right) and end points (left) of our candidate miRNA. The location of alignment hit points is indicated on the y–axis, and the viral *Flavivirus* genome sequences for the capsid protein are indicated on the x–axis. Start points are indicated by diamonds, and the end points are indicated by triangles.

**FIGURE 16:**
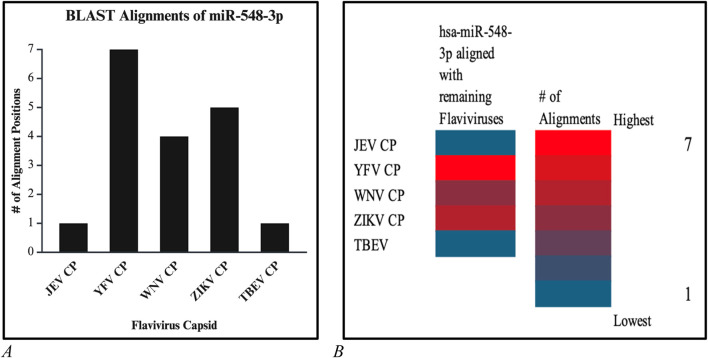
**(A)** Bar graph and **(B)** and heatmap chart that depicted the number of alignment positions that the candidate miRNA displays. Both reveal that the highest number of alignments is 7 (red) and the lowest number of alignments is 1 (blue).

After running series of alignments, our results revealed that hsa-miR-548d-3p (MI0003668) showed complementary sequence structures with the viral genome sequences that are responsible for the nucleocapsid gene of the BVDV and flavivirus capsid protein synthesis. Table 2 shows the details about miR-548d-3p, which includes, name, species, accession number, tissues, sequence, and website. According to BLAST-n, hsa-miR-548d-3p exhibited 100% similarities and showed the highest numbers of alignment positions on the YFV capsid gene (7 locations), as compared to the BVDV NP gene and ZIKV CP gene (Figure 7; 5 locations), and 4 alignment positions on the WNV capsid gene. Figures 8A, B shows the number of alignment positions of hsa-miR-548d-3p on our virus' genome. Table 3 shows all alignment data exhibited by hsa-miR-548d-3p on viral genomes of DENV (Figures 9), HCV, and the other flavivirus members, and Figures 10–16 show the number alignment locations of hsa-miR-548d-3p on the flavivirus family's genome.

**TABLE 2:** Details about the hsa-miR-548-3p sequence.

Name of miRNA	Species	Accession #	Tissue	Sequence	Website
hsa-miR-548-3p	*Homo sapiens* (Human)	MIMAT0003323	Melanoblast	CAAAAACCACAGUUUCUUUUGC	https://mirbase.org/mature/MIMAT0003323?mature_acc=MIMAT0003323

**TABLE 3:** All the alignment data on hsa-miR-548-3p to the capsid genome of all members of the flaviviruses retrieved from NCBI BLASTn, including the name of the genome and the name of the miRNA hsa-miR-548d-3p.

Virus genome	miRNA name and sequence	# Of matches	Score	E-Value	Identities	Query location	Subject location
BVDV Nucleocapsid protein	hsa-miR-548d-3p CAAAAACCACAGUUUCUUUUGC	5	14.4 bits (7)	0.22	7/7 (100%)	Query 11–18	Subject 245–238
			14.4 bits (7)	0.22	7/7 (100%)	Query 15–21	Subject 14–8
			14.4 bits (7)	0.22	7/7 (100%)	Query 16–22	Subject 45–39
			14.4 bits (7)	0.22	7/7 (100%)	Query 13–19	Subject 165–159
			14.4 bits (7)	0.22	7/7 (100%)	Query 3–9	Subject 287–281
HCV Genotype 1a Capsid protein	hsa-miR-548d-3p CAAAAACCACAGUUUCUUUUGC	2	16.3 bits (8)	0.11	8/8 (100%)	Query 2–9	Subject 27–34
			14.4 bits (7)	0.43	7/7 (100%)	Query 13–19	Subject 29–23
HCV Genotype 2 capsid protein	hsa-miR-548d-3p CAAAAACCACAGUUUCUUUUGC	1	20.3 bits (10)	0.003	10/10 (100%)	Query 12–21	Subject 42–33
HCV Genotype 3 capsid protein	has-miR-548d-3p CAAAAACCACAGUUUCUUUUGC0.43	3	20.3 bits (10)	0.007	10/10 (100%)	Query 12–21	Subject 42–33
			16.4 bits (8)	0.11	8/8 (100%)	Query 2–9	Subject 27–34
			14.4 bits (7)	0.43	7/7 (100%)	Query 13–19	Subject 29–23
HCV Genotype 4 capsid protein	hsa-miR-548d-3p CAAAAACCACAGUUUCUUUUGC	2	16.4 bits (8)	0.11	8/8 (100%)	Query 2–9	Subject 27–34
			14.4 bits (7)	0.43	7/7 (100%)	Query 13–9	Subject 29–23
HCV Genotype 5 capsid Protein	has-miR-548d-3p CAAAAACCACAGUUUCUUUUGC	3	20.3 bits (10)	0.007	10/10 (100%)	Query 12–21	Subject 42–33
			16.4 bits (8)	0.11	8/8 (100%)	Query 2–9	Subject 27–34
			14.4 bits (7)	0.43	7/7 (100%)	Query 13–19	Subject 29–23
HCV Genotype 6 capsid protein	hsa-miR-548d-3p CAAAAACCACAGUUUCUUUUGC	2	16.4 bits (8)	0.11	8/8 (100%)	Query 2–9	Subject 27–34
			14.4 bits (7)	0.43	7/7 (100%)	Query 13–19	Subject 29–23
Dengue virus capsid protein	hsa-miR-548d-3p CAAAAACCACAGUUUCUUUUGC	1	14.4 bits (7)	0.19	7/7 (100%)	Query 1–7	Subject 66–60
Dengue virus 1 capsid protein	hsa-miR-548d-3p CAAAAACCACAGUUUCUUUUGC	1	14.4 bits (7)	0.22	7/7 (100%)	Query 8–14	Subject 80–86
Dengue Virus 2 capsid protein	hsa-miR-548d-3p CAAAAACCACAGUUUCUUUUGC	1	14.4 bits (7)	0.25	7/7 (100%)	Query 9–15	Subject 139–133
Dengue Virus 3 capsid protein	hsa-miR-548d-3p CAAAAACCACAGUUUCUUUUGC	1	14.4 bits (7)	0.25	7/7 (100%)	Query 8–14	Subject 80–86
Dengue Virus 4 capsid protein	hsa-miR-548d-3p CAAAAACCACAGUUUCUUUUGC	2	14.4 bits (7)	0.26	7/7 (100%)	Query 1–7	Subject 78–72
			14.4 bits (7)	0.26	7/7 (100%)	Query 14–20	Subject 140–134
Japanese encephalitis virus capsid protein	hsa-miR-548d-3p CAAAAACCACAGUUUCUUUUGC	1	14.4 bits (7)	0.18	7/7 (100%)	Query 10–16	Subject 207–213
Yellow fever virus capsid protein	hsa-miR-548d-3p CAAAAACCACAGUUUCUUUUGC	7	16.4 bits (8)	0.42	8/8 (100%)	Query 13–20	Subject 180–187
			14.4 bits (7)	1.7	7/7 (100%)	Query 2–8	Subject 57–63
			14.4 bits (7)	1.7	7/7 (100%)	Query 3–9	Subject 686–692
			14.4 bits (7)	1.7	7/7 (100%)	Query 15–21	Subject 757–751
			14.4 bits (7)	1.7	7/7 (100%)	Query 12–18	Subject 1729–1735
			14.4 bits (7)	1.7	7/7 (100%)	Query 12–18	Subject 2,298–2,292
			14.4 bits (7)	1.7	7/7 (100%)	Query 1–7	Subject 2,349–2,343
West Nile virus capsid protein	hsa-miR-548d-3p CAAAAACCACAGUUUCUUUUGC	4	14.4 bits (7)	0.16	7/7 (100%)	Query 12–18	Subject 27–33
			14.4 bits (7)	0.16	7/7 (100%)	Query 12–18	Subject 61–55
			14.4 bits (7)	0.16	7/7 (100%)	Query 2–8	Subject 76–82
			14.4 bits (7)	0.16	7/7 (100%)	Query 15–21	Subject 195–201
Zika virus capsid protein	hsa-miR-548d-3p CAAAAACCACAGUUUCUUUUGC	5	16.4 bits (8)	0.10	8/8 (100%)	Query 13–20	Subject 101–94
			14.4 bits (7)	0.41	7/7 (100%)	Query 11–17	Subject 70–76
			14.4 bits (7)	0.41	7/7 (100%)	Query 2–8	Subject 84–90
			14.4 bits (7)	0.41	7/7 (100%)	Query 6–12	Subject 432–439
			14.4 bits (7)	0.41	7/7 (100%)	Query 5–11	Subject 524–530

This Table also includes the number (#) of complementary matches between the miRNA and the viral mRNA genome sequence. The alignment score shows a significantly high score, which indicates a high degree of similar alignments between our miRNA sequence and our virus genome sequence. This table includes the E-value that measures the number of alignments similar found by hsa-miR-548d-3p by chance. The 100% of identity states that the similarity of our sequence alignments has a perfect match. These results included the query locations, which is the location on our miRNA alignment that has the perfect math, while the subject location, which is our mRNA alignment location on our viral genome sequence. On our subject, we see that our miRNA candidate aligns at multiple locations ranging from 14 to 2,349 locations.

## hsa-miR-548d-3p aligned with dengue virus

Our results showed that the hsa-miR-548d-3p sequence is identical to the capsid sequence of dengue virus (DENV) and its four serotypes (Figures 9, 10; Table 1). We found that miR-548-3p has a 100% perfect match on DENV virus genome sequences, as displayed in Table 3. Figures 12A, B show that out of the four serotypes of DENV, 1 out of 5 (20%) showed 2 alignment locations and aligns at 2 nucleotides on the DENV 4 genome at subject locations 78–72 and 140–134.

### hsa-miR-548d-3p alignment with HCV

The alignment analysis also revealed multiple sequence alignments between hsa-miR-548d-3p and the capsid sequence of the HCV virus and its genotypes. Figure 15 shows that out of the six genotypes of HCV, the highest number of alignment locations is observed between our miRNA and HCV genotype 3 and genotype 5 (33.33%) and the second highest number of locations is observed on genotype 1, genotype 4, and genotype 6 (50%).

### hsa-miR-548d-3p aligned with *Flavivirus* capsid gene

Additional alignments were carried out, and it showcased that miR-548d-3p harbors sequence similarities with the capsid genome of the remaining *Flavivirus* members [ZIKV (Figure 7), YFV, WNV, and TBEV]. Figure 15 shows that our candidate miRNA shows similarities on various sections on the flavivirus capsid genome, and Figures 16A, B demonstrate that hsa-miR-548d-3p has the highest number of alignments on the YFV capsid genome (7 alignment locations), 4 alignment positions on the WNV capsid genome, and 5 alignment positions on the ZIKV capsid genome, depicted by this sequence alignment analysis.

## Discussion

miRNAs are a class of small non-coding RNA segments, ranging up to 22 nt long, and serve as possible inhibiting regulators against viral mRNA expression during virus replication (Hasan et al., 2014). A perfect complementary sequence between miRNA and mRNA regions is believed to be sufficient for successful cleavage or degradation of the mRNA sequence, but imperfect alignments may block viral translation (Casal et al., 2004; Hasan et al., 2014). We studied the sequence homology of our miRNA sequence and the flavivirus genome sequences, and we found that after numerous sequence alignments, this study confirms the significant complementary sequence of our candidate miRNA sequence on the flavivirus capsid genome. After careful analysis, we have analyzed that hsa-miR-548d-3p showed identical alignment locations on the capsid gene of DENV 1, 3, and 4 viruses with some minor differences (Figure 11; Table 3). Figure 13 also confirms that hsa-miR-548d-3p also has identical alignment locations on the HCV virus and its genotypes. Hence, those miRNAs are used as antiviral therapeutics; these findings suggest that hsa-miR-548d-3p may be a possible candidate as a universal antiviral therapeutic agent against infections caused by the flavivirus family.

### Alignment data of NCBI BLAST-n

To understand the idea of a good alignment between two sequences, it is necessary to understand the score, E-value (expected value), percentage of identity, and gaps. The bits score indicates how significant the alignment is; the higher the score on the alignment, the better. Observing the expected or, simply, the E-value indicates the significance of an alignment; the lower the E-value signifies, the better the alignment between two sequences. According to Tom Madden, if an alignment has an E-value of 0.05, then the similarities have a 5 in 100 possibility of occurring by chance (Madden, 2013). The percentage (%) of identity signifies how perfect the alignment is between two sequences. Table 3 shows that all the alignments demonstrated are 7/7 or 8/8, which indicates two sequences are 100% similar. According to Fassler and Coopet (2008), the percentage of identity of 100% means that the nucleotides of the subject sequence are identical to the reference sequence or the query at every position of the alignment.

### Using miRBase for the prediction of miRNA targets

Many bioinformatics tools were developed for biogenesis and to help biologists investigate miRNA biology. Among these tools, miRBase (https://mirbase.org/) is the most widely used software program (Chen, L., et al., 2019), which was developed in the year 2002 (Chen, L., et al., 2019). Later, the name changed from “the microRNA Registry” provided molecular researchers with stable and unique gene names for their novel miRNA discoveries and storages of miRNA sequences (Kozomara and Griffiths-Jones, 2010). miRBase is a primary repository database for retrieving data on miRNA and has three main functions: 1) provides confidential services for independent assignment of miRNA genes, 2) sequences provide miRNA data, annotation, references, and links to other published miRNAs, and 3) provides miRNA target pipelines for the prediction of the target (Griffiths-Jones et al., 2007; Griffiths-Jones, 2006). miRBase also allows searching published pre-miRNA and mature miRNA sequences, in addition to readily available annotation and sequence data that are available for download (Luna Buitrago et al., 2023). Overall, miRBase provides scientists a variety of data on miRNAs when obtaining sequences that include the accession number, symbols, description, and gene family.

### Using miRBase and BLASTn

One of the important function of miRbase is to provide a microRNA target pipeline for the prediction of targets for all published animal miRNAs (Griffiths-Jones et al., 2007; Griffiths-Jones, 2006), and all miRNA sequences from this database revealed interactions against 3′-untranslated regions, which are predicted from all available species (Griffiths-Jones, 2006).

We used BLAST to perform alignments for its potential to detect similar regions within parts of long sequences. It is a fast, sensitive, and accurate tool for analyzing sequences for alignments (Altschul, et al., 1990; Lobo, 2008). The reason that miRBase does not run alignment analysis was BLAST was used when searching for the right miRNA sequences over 2,600 miRNAs. Additionally, BLAST was used because it is the most widely used software package in bioinformatics research due to its main function of comparing sequence(s) of interest (Stover and Cavalcanti, 2017).

## Roles of hsa-miR-548d-3p in humans

hsa-miR-548d-3p is a mature miRNA that is found in primates and comprises over 69 identified miRNAs that are presented in all human chromosomes, but also as a more poorly conserved miRNA (Ramos-Sanchez et al., 2022), it demonstrates to enhance cell proliferation and inhibit apoptosis in breast cancer (Souza et al., 2016; Souza et al., 2021). Functions can include many biological processes, such as signaling pathways like MAPK, phosphatidylinositol (P13K), p53, B-cell receptor, T-cell receptor, TGF-beta, PPAR, calcium, and insulin signaling pathways, and in human tumorigenesis, such as colorectal cancer, glioma, and non-small cell lung cancer (Ramox-Sanchez, 2022; Liang et al., 2012). hsa-miR-548d-3p is proven to be involved in homeostasis of stress damage, and metabolic and survival pathways for cell proliferation (Cannataro and Cione, 2019; Maiorino et al., 2015). In an experiment done by Rooda L. et al., their results indicated that hsa-miR-548d-3p and its family may play additional roles in humans, such as in ovarian follicle activation, development, granulosa cell differentiation, and proliferation (Rooda et al., 2021).

### Bovine viral diarrhea virus as a model for flaviviruses

Prestiviruses are more closely related to HCV than the classical flaviviruses and have been used as surrogate models for HCV (Tellinghuisen et al., 2006; Lackner et al., 2004) to test *in vitro* infectivity (Durantel et al., 2004). According to Chen et. al. (2022), as one of the most characterized members of the Flaviviridae family, BVDV serves as a good model system to study flaviviruses and has primarily been used as a surrogate model for HCV in identification and characterization of antiviral agents (Finkielsztein et al., 2010). This approach leverages the similarities between BVDV and HCV to develop and test potential treatments for HCV more effectively. Lai et al. (2000) stated that both viruses BVDV and HCV utilize the IRES within the 5′ Untranslated Region (UTR) necessary for translation of viral polyprotein, while NS3 proteases of both viruses require NS4A as a cofactor for polyprotein processing.

### Limitations

This was a pure computer-based study using bioinformatic tools to showcase possible miRNA–mRNA sequence similarities. Due to pestiviruses like the BVDV, which is used as a surrogate model for studying HCV virus, we hypothesized that if we can utilize the BVDV genome sequence as our test subject, then we could find a possible universal miRNA-based antiviral therapeutic for the family of flaviviruses. Based on our results and Table 3 and Figures 10–16, we found hsa-miR-548d-3p as a possible candidate due to its perfect match with the genome of all our viruses rather than just one. Again, this is a full bioinformatic-based analytical study, where *in vivo* lab equipment was not used. Hence, the results are not considered final until proved using *in vivo* experimentation.

## Conclusion

After performing a series of sequence alignments, we predicted hsa-miR-548d-3p, a mature miRNA sequence, as a potential candidate to target flaviviruses showing perfect alignments with BVDV; HCV genotype 1a, 2, 3, 4, 5, and 6; DENV serotype 1, 2, 3, and 4; JENV; WNV; ZIKA; and TBEV. Although more detailed *in vitro* and *in vivo* studies are required to utilize hsa-miR-548d-3p as an antiviral therapeutic, this study may be considered a first step to develop a new type of miRNA treatment against a range of viruses within the Flaviviridae family. This study also recognizes that the BVDV may not be the surrogate model for only HCV virus but can also prove to be a good model system for antiviral therapeutic studies against other members of the Flaviviridae family.

## Data Availability

The datasets presented in this study can be found in online repositories. The names of the repository/repositories and accession number(s) can be found in the article/supplementary material.

## References

[B2] Altschul S. F. Gish W. Miller W. Myers E. W. Lipman D. J. (1990). Basic local alignment search tool. J. Mol. Biol. 215 (3), 403–410. 10.1006/jmbi.1990.9999 2231712

[B3] Ardekani A. M. Naeini M. M. (2010). The role of MicroRNAs in human diseases. Avicenna J. Med. Biotechnol. 2 (4), 161–179. 23407304 PMC3558168

[B4] Babiker A. Jeudy J. Kligerman S. Khambaty M. Shah A. Bagchi S. (2017). Risk of cardiovascular disease due to chronic hepatitis C infection: a review. J. Clin. Transl. hepatology 5 (4), 1–20. 10.14218/jcth.2017.00021 PMC5719192 29226101

[B5] Baek D. Villén J. Shin C. Camargo F. D. Gygi S. P. Bartel D. P. (2008). The impact of microRNAs on protein output. Nature 455 (7209), 64–71. 10.1038/nature07242 18668037 PMC2745094

[B6] Barrows N. J. Campos R. K. Liao K. C. Prasanth K. R. Soto-Acosta R. Yeh S. C. (2018). Biochemistry and molecular biology of flaviviruses. Chem. Rev. 118 (8), 4448–4482. 10.1021/acs.chemrev.7b00719 29652486 PMC5937540

[B7] Basarab M. Bowman C. Aarons E. J. Cropley I. (2016). Zika virus. Bmj 352, i1049. 10.1136/bmj.i1049 26921241

[B8] Becher P. Orlich M. Thiel H. J. (1998). Complete genomic sequence of border disease virus, a pestivirus from sheep. J. virology 72 (6), 5165–5173. 10.1128/jvi.72.6.5165-5173.1998 9573288 PMC110089

[B9] Benzarti E. Linden A. Desmecht D. Garigliany M. (2019). Mosquito-borne epornitic flaviviruses: an update and review. J. General Virology 100 (2), 119–132. 10.1099/jgv.0.001203 30628886

[B10] Cannataro R. Cione E. (2019). Antioxidant and microRNAs: an applied overview. J. Microbiol. Biotechnol. Rep.

[B11] Casal M. L. Dambach D. M. Meister T. Jezyk P. F. Patterson D. F. Henthorn P. S. (2004). Familial glomerulonephropathy in the bullmastiff. Veterinary pathol. 41 (4), 319–325. 10.1354/vp.41-4-319 15232131

[B12] Chen L. Heikkinen L. Wang C. Yang Y. Sun H. Wong G. (2019). Trends in the development of miRNA bioinformatics tools. Briefings Bioinforma. 20 (5), 1836–1852. 10.1093/bib/bby054 PMC7414524 29982332

[B13] Chen N. Liu Y. Bai T. Chen J. Zhao Z. Li J. (2022). Quercetin inhibits Hsp70 blocking of bovine viral diarrhea virus infection and replication in the early stage of virus infection. Viruses 14 (11), 2365. 10.3390/v14112365 36366463 PMC9692758

[B14] Chi S. Chen S. Jia W. He Y. Ren L. Wang X. (2022). Non-structural proteins of bovine viral diarrhea virus. Virus genes 58 (6), 491–500. 10.1007/s11262-022-01914-8 35614328 PMC9131992

[B15] Conde J. N. Silva E. M. Barbosa A. S. Mohana-Borges R. (2017). The complement system in flavivirus infections. Front. Microbiol. 8, 213. 10.3389/fmicb.2017.00213 28261172 PMC5306369

[B16] de Oliveira Figueiredo P. Stoffella-Dutra A. G. Barbosa Costa G. Silva de Oliveira J. Dourado Amaral C. Duarte Santos J. (2020). Re-emergence of yellow fever in Brazil during 2016-2019: challenges, lessons learned, and perspectives. Viruses 12 (11), 1233. 10.3390/v12111233 33143114 PMC7692154

[B17] Duncan J. D. Urbanowicz R. A. Tarr A. W. Ball J. K. (2020). Hepatitis C virus vaccine: challenges and prospects. Vaccines 8 (1), 90. 10.3390/vaccines8010090 32079254 PMC7157504

[B18] Durantel D. Carrouée-Durantel S. Branza-Nichita N. Dwek R. A. Zitzmann N. (2004). Effects of interferon, ribavirin, and iminosugar derivatives on cells persistently infected with noncytopathic bovine viral diarrhea virus. Antimicrob. agents Chemother. 48 (2), 497–504. 10.1128/aac.48.2.497-504.2004 14742201 PMC321564

[B19] Fassler J. Cooper P. (2008). “BLAST glossary. 2011 jul 14,” in BLAST® help (Bethesda (MD): National Center for Biotechnology Information US). Available at: https://www.ncbi.nlm.nih.gov/books/NBK62051/.

[B20] Finkielsztein L. M. Moltrasio G. Caputto M. Castro E. Cavallaro L. Moglioni A. (2010). What is known about the antiviral agents active against bovine viral diarrhea virus (BVDV)? Curr. Med. Chem. 17 (26), 2933–2955. 10.2174/092986710792065036 20858174

[B21] Finnegan E. F. Pasquinelli A. E. (2013). MicroRNA biogenesis: regulating the regulators. Crit. Rev. Biochem. Mol. Biol. 48 (1), 51–68. 10.3109/10409238.2012.738643 23163351 PMC3557704

[B22] Fu G. Brkić J. Hayder H. Peng C. (2013). MicroRNAs in human placental development and pregnancy complications. Int. J. Mol. Sci. 14 (3), 5519–5544. 10.3390/ijms14035519 23528856 PMC3634453

[B23] Fulton R. W. Purdy C. W. Confer A. W. Saliki J. T. Loan R. W. Briggs R. E. (2000). Bovine viral diarrhea viral infections in feeder calves with respiratory disease: interactions with Pasteurella spp., parainfluenza-3 virus, and bovine respiratory syncytial virus. Can. J. veterinary Res. = Revue Can. de recherche veterinaire 64 (3), 151–159. PMC1189606 10935880

[B24] Giménez-Richarte Á. Ortiz de Salazar M. I. Giménez-Richarte M. P. Collado M. Fernández P. L. Clavijo C. (2022). Transfusion-transmitted arboviruses: update and systematic review. PLOS Neglected Trop. Dis. 16 (10), e0010843. 10.1371/journal.pntd.0010843 PMC9578600 36201547

[B25] Griffiths-Jones S. (2006). miRBase: microRNA sequences, targets and gene nomenclature. Nucleic acids Res. 34 (Database issue), D140–D144. 10.1093/nar/gkj112 16381832 PMC1347474

[B26] Griffiths-Jones S. Saini H. K. van Dongen S. Enright A. J. (2007). miRBase: tools for microRNA genomics. Nucleic acids Res. 36 (Suppl. l_1), D154–D158. 10.1093/nar/gkm952 17991681 PMC2238936

[B28] Ha M. Kim V. N. (2014). Regulation of microRNA biogenesis. Nat. Rev. Mol. cell Biol. 15 (8), 509–524. 10.1038/nrm3838 25027649

[B29] Hamel R. Dejarnac O. Wichit S. Ekchariyawat P. Neyret A. Luplertlop N. (2015). Biology of Zika virus infection in human skin cells. J. virology 89 (17), 8880–8896. 10.1128/jvi.00354-15 26085147 PMC4524089

[B30] Harada T. Tautz N. Thiel H. J. (2000). E2-p7 region of the bovine viral diarrhea virus polyprotein: processing and functional studies. J. virology 74 (20), 9498–9506. 10.1128/jvi.74.20.9498-9506.2000 11000219 PMC112379

[B31] Hasan M. M. Akter R. Ullah M. S. Abedin M. J. Ullah G. M. A. Hossain M. Z. (2014). A computational approach for predicting role of human microRNAs in MERS‐CoV genome. Adv. Bioinforma. 2014 (1), 1–8. 10.1155/2014/967946 PMC4283225 25610462

[B32] Hills S. L. Fischer M. Petersen L. R. (2017). Epidemiology of Zika virus infection. J. Infect. Dis. 216 (Suppl. l_10), S868–S874. 10.1093/infdis/jix434 29267914 PMC5853392

[B33] Hu T. Wu Z. Wu S. Chen S. Cheng A. (2021). The key amino acids of E protein involved in early flavivirus infection: viral entry. Virology J. 18 (1), 136. 10.1186/s12985-021-01611-2 34217298 PMC8254458

[B90] Hause B. M Pillatzki A. Clement T. Bragg T. Ridpath J. Chase C. C. L. (2021). Persistent infection of American bison (Bison bison) with bovine viral diarrhea virus and bosavirus. Vet. Microbiol. 252, 108949. 10.1016/j.vetmic.2020.108949 33338948

[B34] Isken O. Baroth M. Grassmann C. W. Weinlich S. Ostareck D. H. Ostareck-Lederer A. (2007). Nuclear factors are involved in hepatitis C virus RNA replication. Rna 13 (10), 1675–1692. 10.1261/rna.594207 17684232 PMC1986813

[B35] Jackova A. Novackova M. Pelletier C. Audeval C. Gueneau E. Haffar A. (2008). The extended genetic diversity of BVDV-1: typing of BVDV isolates from France. Veterinary Res. Commun. 32 (1), 7–11. 10.1007/s11259-007-9012-z 17657577

[B36] Julander J. G. Shafer K. Smee D. F. Morrey J. D. Furuta Y. (2009). Activity of T-705 in a hamster model of yellow fever virus infection in comparison with that of a chemically related compound, T-1106. Antimicrob. agents Chemother. 53 (1), 202–209. 10.1128/aac.01074-08 18955536 PMC2612161

[B37] Junglen S. Kopp A. Kurth A. Pauli G. Ellerbrok H. Leendertz F. H. (2009). A new flavivirus and a new vector: characterization of a novel flavivirus isolated from uranotaenia mosquitoes from a tropical rain forest. J. virology 83 (9), 4462–4468. 10.1128/jvi.00014-09 19224998 PMC2668441

[B38] Khodakaram-Tafti A. Farjanikish G. H. (2017). Persistent bovine viral diarrhea virus (BVDV) infection in cattle herds. Iran. J. veterinary Res. 18 (3), 154–163. PMC5674437 29163643

[B39] Kosinova E. Psikal I. Robesova B. Kovarcik K. (2007). Real-time PCR for quantitation of bovine viral diarrhea virus RNA using SYBR Green I fluorimetry. VETERINARNI MEDICINA-PRAHA- 52 (6), 253–261. 10.17221/1882-vetmed

[B40] Kozomara A. Griffiths-Jones S. (2010). miRBase: integrating microRNA annotation and deep-sequencing data. Nucleic acids Res. 39 (Suppl. l_1), D152–D157. 10.1093/nar/gkq1027 21037258 PMC3013655

[B41] Lackner T. Müller A. Pankraz A. Becher P. Thiel H. J. Gorbalenya A. E. (2004). Temporal modulation of an auto protease is crucial for replication and pathogenicity of an RNA virus. J. virology 78 (19), 10765–10775. 10.1128/jvi.78.19.10765-10775.2004 15367643 PMC516412

[B42] Lai V. C. Zhong W. Skelton A. Ingravallo P. Vassilev V. Donis R. O. (2000). Generation and characterization of a hepatitis C virus NS3 protease-dependent bovine viral diarrhea virus. J. virology 74 (14), 6339–6347. 10.1128/jvi.74.14.6339-6347.2000 10864644 PMC112140

[B43] Laureti M. Narayanan D. Rodriguez-Andres J. Fazakerley J. K. Kedzierski L. (2018). Flavivirus receptors: diversity, identity, and cell entry. Front. Immunol. 9, 2180. 10.3389/fimmu.2018.02180 30319635 PMC6168832

[B44] Li F. Wang Y. Yu L. Cao S. Wang K. Yuan J. (2015). Viral infection of the central nervous system and neuroinflammation precede blood-brain barrier disruption during Japanese encephalitis virus infection. J. virology 89 (10), 5602–5614. 10.1128/jvi.00143-15 25762733 PMC4442524

[B45] Li Y. Wang J. Kanai R. Modis Y. (2013). Crystal structure of glycoprotein E2 from bovine viral diarrhea virus. Proc. Natl. Acad. Sci. 110 (17), 6805–6810. 10.1073/pnas.1300524110 23569276 PMC3637714

[B46] Liang T. Guo L. Liu C. (2012). Genome‐wide analysis of mir‐548 gene family reveals evolutionary and functional implications. BioMed Res. Int. 2012 (1), 1–8. 10.1155/2012/679563 PMC3468316 23091353

[B47] Lobo I. (2008). Basic local alignment search tool (BLAST). Nat. Educ. 1 (1).

[B48] Luna Buitrago D. Lovering R. C. Caporali A. (2023). Insights into online microRNA bioinformatics tools. Non-coding RNA 9 (2), 18. 10.3390/ncrna9020018 36960963 PMC10037614

[B49] Madden T. (2013). The BLAST sequence analysis tool. NCBI Handb. 2 (5), 425–436.

[B50] Maiorino M. Bosello-Travain V. Cozza G. Miotto G. Roveri A. Toppo S. (2015). Understanding mammalian glutathione peroxidase 7 in the light of its homologs. Free Radic. Biol. Med. 83, 352–360. 10.1016/j.freeradbiomed.2015.02.017 25724691

[B51] Mari V. L. Losurdo M. Lucente M. S. Lorusso E. Elia G. Martella V. (2016). Multiplex real-time RT-PCR assay for bovine viral diarrhea virus type 1, type 2 and HoBi-like pestivirus. J. virological methods 229, 1–7. 10.1016/j.jviromet.2015.12.003 PMC7113868 26709100

[B52] Maurer K. Krey T. Moennig V. Thiel H. J. Rümenapf T. (2004). CD46 is a cellular receptor for bovine viral diarrhea virus. J. virology 78 (4), 1792–1799. 10.1128/jvi.78.4.1792-1799.2004 14747544 PMC369467

[B53] Merwaiss F. Czibener C. Alvarez D. E. (2019). Cell-to-cell transmission is the main mechanism supporting bovine viral diarrhea virus spread in cell culture. J. Virology 93 (3), 017766-18–e11128. 10.1128/jvi.01776-18 PMC6340029 30404802

[B54] Murray C. L. Marcotrigiano J. Rice C. M. (2008). Bovine viral diarrhea virus core is an intrinsically disordered protein that binds RNA. J. virology 82 (3), 1294–1304. 10.1128/jvi.01815-07 18032507 PMC2224441

[B55] Musso D. Gubler D. J. (2016). Zika virus. Clin. Microbiol. Rev. 29 (3), 487–524. 10.1128/cmr.00072-15 27029595 PMC4861986

[B56] Musso D. Nhan T. X. (2015). Emergence of Zika virus. Clin. Microbiol. 4, 222. 10.4172/2327-5073.1000222 32260029

[B57] Mutebi J. P. Rijnbrand R. C. A. Wang H. Ryman K. D. Wang E. Fulop L. D. (2004). Genetic relationships and evolution of genotypes of yellow fever virus and other members of the yellow fever virus group within the Flavivirus genus based on the 3′ noncoding region. J. virology 78 (18), 9652–9665. 10.1128/jvi.78.18.9652-9665.2004 15331698 PMC515011

[B58] Neill J. D. (2013). Molecular biology of bovine viral diarrhea virus. Biologicals 41 (1), 2–7. 10.1016/j.biologicals.2012.07.002 22884672

[B59] Nijmeijer B. M. Koopsen J. Schinkel J. Prins M. Geijtenbeek T. B. (2019). Sexually transmitted hepatitis C virus infections: current trends, and recent advances in understanding the spread in men who have sex with men. J. Int. AIDS Soc. 22 (l), e25348. 10.1002/jia2.25348 31468692 PMC6715947

[B60] Niskanen R. Lindberg A. Larsson B. Alenius S. (2000). Lack of virus transmission from bovine viral diarrhoea virus infected calves to susceptible peers. Acta Veterinaria Scand. 41 (1), 93–99. 10.1186/bf03549659 PMC7996413 10920480

[B61] O'Brien J. Hayder H. Zayed Y. Peng C. (2018). Overview of microRNA biogenesis, mechanisms of actions, and circulation. Front. Endocrinol. 9, 402. 10.3389/fendo.2018.00402 PMC6085463 30123182

[B62] Perera-Lecoin M. Meertens L. Carnec X. Amara A. (2013). Flavivirus entry receptors: an update. Viruses 6 (1), 69–88. 10.3390/v6010069 24381034 PMC3917432

[B63] Peterhans E. Jungi T. W. Schweizer M. (2003). BVDV and innate immunity. Biologicals 31 (2), 107–112. 10.1016/s1045-1056(03)00024-1 12770540

[B64] Pierson T. C. Diamond M. S. (2020). The continued threat of emerging flaviviruses. Nat. Microbiol. 5 (6), 796–812. 10.1038/s41564-020-0714-0 32367055 PMC7696730

[B65] Pierson T. C. Kielian M. (2013). Flaviviruses: braking the entering. Curr. Opin. virology 3 (1), 3–12. 10.1016/j.coviro.2012.12.001 PMC3601797 23352692

[B66] Qian Q. Xu R. Wang Y., (2022). “The NS4A protein of classical swine fever virus suppresses RNA silencing in mammalian cells.” J. Virology, 96(15), 018744-e1921. 10.1128/jvi.01874-21 PMC9364796 35867575

[B67] Ramos-Sanchez E. M. Reis L. C. Souza M. A. Muxel S. M. Santos K. R. Lagos D. (2022). miR-548d-3p is up-regulated in human visceral leishmaniasis and suppresses parasite growth in macrophages. Front. Cell. Infect. Microbiol. 12, 826039. 10.3389/fcimb.2022.826039 35265535 PMC8900537

[B68] Ranganathan K. Sivasankar V. (2014). MicroRNAs-Biology and clinical applications. J. Oral Maxillofac. Pathology 18 (2), 229–234. 10.4103/0973-029x.140762 PMC4196292 25328304

[B69] Reed K. E. Gorbalenya A. E. Rice C. M. (1998). The NS5A/NS5 proteins of viruses from three genera of the family flaviviridae are phosphorylated by associated serine/threonine kinases. J. virology 72 (7), 6199–6206. 10.1128/jvi.72.7.6199-6206.1998 9621090 PMC110437

[B70] Rooda I. Kaselt B. Liivrand M. Smolander O. P. Salumets A. Velthut-Meikas A. (2021). Hsa-mir-548 family expression in human reproductive tissues. BMC Genomic Data 22, 40–13. 10.1186/s12863-021-00997-w 34625017 PMC8501715

[B71] Schweizer M. Peterhans E. (2001). Noncytopathic bovine viral diarrhea virus inhibits double-stranded RNA-induced apoptosis and interferon synthesis. J. Virology 75 (10), 4692–4698. 10.1128/jvi.75.10.4692-4698.2001 11312340 PMC114223

[B72] Selbach M. Schwanhäusser B. Thierfelder N. Fang Z. Khanin R. Rajewsky N. (2008). Widespread changes in protein synthesis induced by microRNAs. nature 455 (7209), 58–63. 10.1038/nature07228 18668040

[B73] Simmons C. P. Farrar J. J. van Vinh Chau N. Wills B. (2012). Dengue. N. Engl. J. Med. 366 (15), 1423–1432. 10.1056/nejmra1110265 22494122

[B74] Skalsky R. L. Cullen B. R. (2010). Viruses, microRNAs, and host interactions. Annu. Rev. Microbiol. 64, 123–141. 10.1146/annurev.micro.112408.134243 20477536 PMC3621958

[B75] Song Z. Q. Hao F. Min F. Ma Q. Y. Liu G. D. (2001). Hepatitis C virus infection of human hepatoma cell line 7721 *in vitro* . World J. Gastroenterology 7 (5), 685. 10.3748/wjg.7.685 PMC4695574 11819854

[B76] Souza M. A. Almeida T. M. Castro M. C. A. Oliveira-Mendes A. P. Almeida A. F. Oliveira B. C. (2016). American tegumentary leishmaniasis: mRNA expression for Th1 and Treg mediators are predominant in patients with recent active disease. Immunobiology 221 (2), 253–259. 10.1016/j.imbio.2015.08.009 26572279

[B77] Souza M. D. A. Ramos-Sanchez E. M. Muxel S. M. Lagos D. Reis L. C. Pereira V. R. A. (2021). miR-548d-3p alters parasite growth and inflammation in leishmania (Viannia) braziliensis infection. Front. Cell. Infect. Microbiol. 11, 687647. 10.3389/fcimb.2021.687647 34178725 PMC8224172

[B78] Stover N. A. Cavalcanti A. R. (2017). Using NCBI BLAST. Curr. Protoc. Essent. Lab. Tech. 14 (1), 11–1. 10.1002/cpet.8

[B79] Suzich J. A. Tamura J. K. Palmer-Hill F. Warrener P. Grakoui A. Rice C. M. (1993). Hepatitis C virus NS3 protein polynucleotide-stimulated nucleoside triphosphatase and comparison with the related pestivirus and flavivirus enzymes. J. virology 67 (10), 6152–6158. 10.1128/jvi.67.10.6152-6158.1993 8396675 PMC238037

[B80] Tellinghuisen T. L. Paulson M. S. Rice C. M. (2006). The NS5A protein of bovine viral diarrhea virus contains an essential zinc-binding site similar to that of the hepatitis C virus NS5A protein. J. virology 80 (15), 7450–7458. 10.1128/jvi.00358-06 16840325 PMC1563740

[B81] Turtle L. Griffiths M. J. Solomon T. (2012). Encephalitis caused by flaviviruses. QJM Mon. J. Assoc. Physicians 105 (3), 219–223. 10.1093/qjmed/hcs013 PMC3285924 22367423

[B82] van den Elsen K. Quek J. P. Luo D. (2021). Molecular insights into the flavivirus replication complex. Viruses 13 (6), 956. 10.3390/v13060956 34064113 PMC8224304

[B83] van Leur S. W. Heunis T. Munnur D. Sanyal S. (2021). Pathogenesis and virulence of flavivirus infections. Virulence 12 (1), 2814–2838. 10.1080/21505594.2021.1996059 34696709 PMC8632085

[B84] Waggoner J. J. Rojas A. Pinsky B. A. (2018). Yellow fever virus: diagnostics for a persistent arboviral threat. J. Clin. Microbiol. 56 (10), e00827-18–e01128. 10.1128/jcm.00827-18 30021822 PMC6156298

[B86] Warrener P. Collett M. S. (1995). Pestivirus NS3 (p80) protein possesses RNA helicase activity. J. virology 69 (3), 1720–1726. 10.1128/jvi.69.3.1720-1726.1995 7853509 PMC188775

[B88] World Health Organization (2019). Japanese encephalitis. World Health Organization. Available at: https://www.who.int/news-room/fact-sheets/detail/japanese-encephalitis.

[B89] Xue W. Mattick D. Smith L. Maxwell J. (2009). Fetal protection against bovine viral diarrhea virus types 1 and 2 after the use of a modified-live virus vaccine. Can. J. Veterinary Res. 73 (4), 292–297. PMC2757710 20046631

